# Low adherence to influenza vaccination campaigns: is the H1N1 virus pandemic to be blamed?

**DOI:** 10.1186/1824-7288-37-54

**Published:** 2011-11-10

**Authors:** Valeria Trivellin, Vera Gandini, Luigi Nespoli

**Affiliations:** 1 UO Pediatria, Ospedale "Filippo Del Ponte", Varese, Italy

## Abstract

**Background:**

Over the last few months, debates about the handling of the influenza virus A (H1N1) pandemic took place, in particular regarding the change of the WHO pandemic definition, economic interests, the dramatic communication style of mass media. The activation of plans to reduce the virus diffusion resulted in an important investment of resources. Were those investments proportionate to the risk? Was the pandemic overrated? The workload of the Pediatric Emergency Room (P.E.R.) at a teaching hospital in Varese (Northern Italy) was investigated in order to evaluate the local diffusion and severity of the new H1N1 influenza epidemic.

**Discussion:**

A 100% increase of the number of P.E.R. visits, particularly for influenza-like illness, was recorded during weeks 42-46 of 2009 (October, 17 to November, 2); the low rate of hospitalization and the mild presentation of the infection gave rise to the conclusion that the pandemic risk was overrated. Mass media communications concerning the new virus created a disproportionate fear in the population that significantly enhanced the burden of cares at the hospital. In the absence of generally implemented measures for etiological diagnosis, the actual incidence of the H1N1 infection could not be estimated. Virus identification, in fact, was limited to children showing severe symptoms after consultancy with an infectious disease specialist. The alarming nature of the communication campaign and the choice to limit etiologic diagnosis to severe cases created a climate of uncertainty which significantly contributed to the massive admissions to the P.E.R..

**Summary:**

The communication strategy adopted by the mass media was an important element during the pandemic: the absence of clarity contributed to the spread of a pandemic phobia that appeared to result more from the sensationalism of the campaign than from infection with the novel influenza A variant of human, avian, swine origin virus. One relevant effect of the media coverage was the extremely low adherence rate to the vaccination campaign for the 2009-2010 and 2010-2011, especially among the high- risk population and health care workers. One positive consequence was, however, the spread of preventive hygiene measures, such as hand washing.

## Background

Recently, Giovanni Rezza, Director of the Italian National Institute of Health's Infectious Disease Department, reported a low rate of adherence to the last influenza vaccination campaign, above all from at risk people. Can this be considered one effect of the H1N1 pandemic? On June 11, 2009 the World Health Organization declared the first pandemic of the 21st century, caused by a novel influenza A virus. Debates about the handling of this pandemic immediately emerged: the change of the WHO pandemic definition, the economic and commercial interests about the vaccines and antiviral drugs, mass media communication that described the new influenza as a global, imminent worldwide disaster. Particularly, the European Parliament repeatedly attacked WHO with regard to the inappropriate handling of the crisis. [1-3] Health systems, at both national and local level, activated the response plans prepared to contain the pandemic influenza with a considerable economical investment in order to face the expected danger. In Italy, from April 28, 2009 the Health Ministry activated a surveillance system (Influnet) that detected approximately 5.600.000 influenza-like illnesses (I.L.I.) (as of May 9, 2009), with almost 2,000 cases laboratory-confirmed cases from May to October 2009. A total of 1,100 patients were hospitalised for serious conditions. Of these 532 were admitted to intensive care units. Two hundred and sixty deaths due to influenza were reported. [4]

Debates emerged about the overrated pandemic and continued to the present time, when data coming from surveillance systems showed that the global pandemic wasn't different from an epidemic influenza. Indeed the infection rate reached was 13/1,000 inhabitants, whereas during the 2004-2005 winter season the epidemic influenza incidence exceeded the value of 14/1,000. [5]

To determine the effective relevance and costs of this pandemic for the health system, the activity of the P.E.R. during the H1N1 pandemic was investigated. A visit was considered to for Influenza-Like Illness (I.L.I.) when the chief complaint entered at the triage included fever that was present in 88% - 95% of the patients, even in the absence of other symptoms. [6-10]

## Results

**Figure 1:**
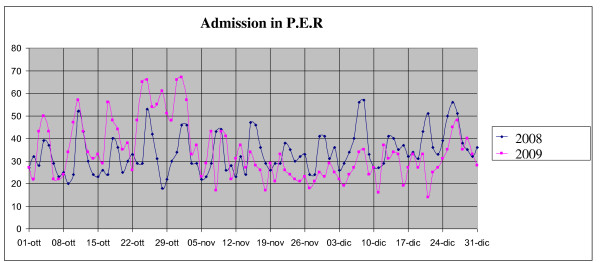
**Mean daily admissions to PER in the 4^th ^quarter of 2008 and 2009**.

**Figure 2:**
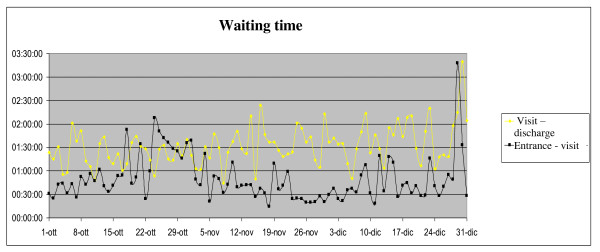
**Accesses to PER: mean waiting time per patient PER in the 4^th ^quarter of 2009**.

From October 24 to November 2, 2009 (weeks 43-44) the average daily access to P.E.R. was 59 patients, almost twice the number of patients normally admitted to P.E.R. in the corresponding period of the previous year (Figure 1). In the same period (Figure 2) the patients' average waiting time was increased, but the time per medical remained unchanged.

**Figure 3:**
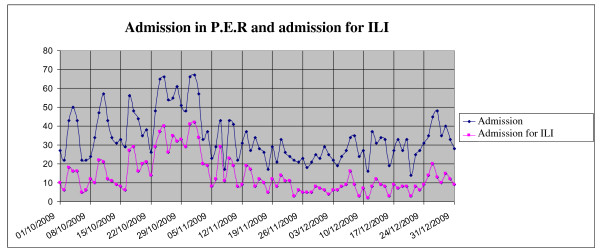
**Mean daily accesses to PER and mean daily accesses with ILI symptoms**.

**Figure 4:**
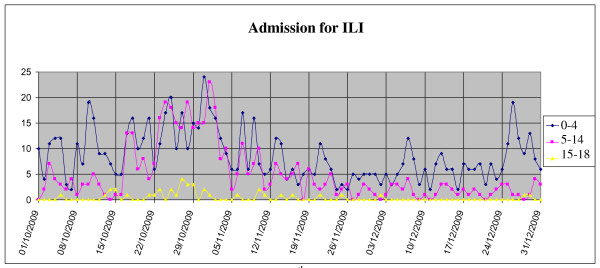
**Mean daily admissions to PER in the 4^th ^quarter of 2009: distribution by age groups**.

**Figure 5:**
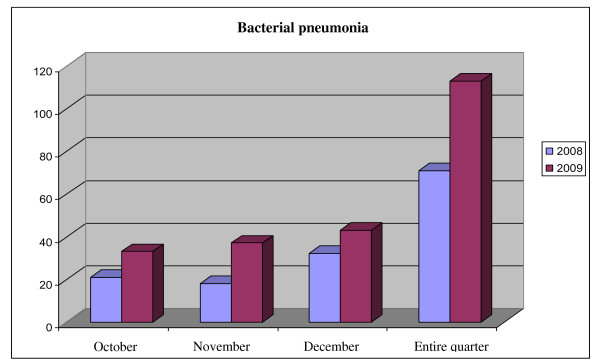
**Cases of bacterial pneumonia diagnosed in PER in the 4th quarter of 2008 and 2009**.

As shown in Figure 3, from October 1 to December 31 2009, 1.284 patients came for I.L.I., in accordance with previously defined criteria: 719 (56%) during the peak of the epidemic weeks 42-46 of 2009. No differences in hospitalization rates were observed with regard to 2009 vs. 2008: patients presenting in the P.E.R. in the fourth quarter of 2009, 17% were hospitalized in 2009, 20% in 2008. During the peak of the pandemic (weeks 43-44), the hospitalization rate was 10%. The peak period was characterized by an increased admittance of children aged 5 to 14 years who presented with I.L.I.. These represented 45% of visits vs. 23% that characterized the rest of the year (Figure 4). Since it is well documented that viral infection can lead to bacterial superinfection [11], the incidence of bacterial pneumonia diagnosed in P.E.R. from October to December in the years 2008 and 2009 was evaluated. One hundred and thirteen cases of bacterial pneumonia were diagnosed during the last week of October and the first of November, vs. 71 cases in the previous year. ( Figure 5).

**Figure 6:**
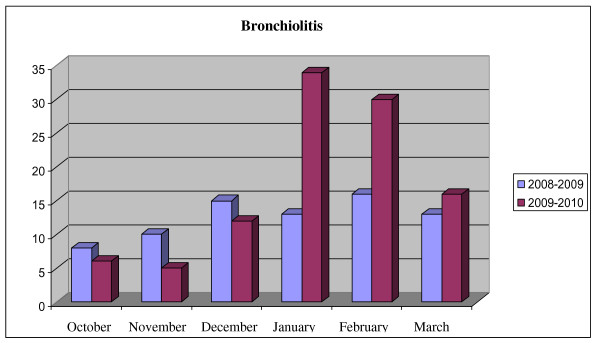
**Cases of bronchiolitis diagnosed in PER in the six months winter periods of 2008-2009 and 2009-2010**.

At a difference with other European countries where the impact of the 2009 influenza pandemic on the pattern of respiratory virus epidemics was demonstrated [12], the incidence of bronchiolitis in the fourth quarter of 2009 was lower with respect to the same period of 2008. An increased incidence of bronchiolitis was instead recorded in the first quarter of 2010. (Figure 6). As compared to 2008, reduced numbers of P.E.R. accesses were recorded from November 15 to December 31, 2009 (Figure 1).

## Conclusions

Influenza A H1N1: a real or a mass media induced epidemic? Certainly the recent influenza epidemic represented a significant care burden for the P.E.R., as evidenced by the excess of visits. It may be concluded that the severity of the novel influenza was overrated. This is confirmed by the observed low rate of hospitalizations, the patients' mild chief complaints and the lack of mortality in the observed territory. The alarm generated by mass-media brought to medical attention slightly ill patients that normally would have turned to the family paediatrician. The reiterated presentation of severe and fatal cases by media generated the idea that novel triple reassortant influenza A virus was indeed responsible of severe pathologies requiring medical attention in the hospital setting. The mortality rate, in fact, effectively gave a direct message that increased fear and uncertainty in the population. One of the negative consequences of this unjustified fear was the low adherence to the 2009 and 2010 influenza vaccination campaigns, especially among the at risk population. The discordance between the alarming forecasts of the mass media and the observed behavior of the epidemic produced a climate of uncertainty both among the general population and healthcare workers who are essential to promote the vaccination campaign.

In our context the policy to limit the etiologic diagnosis to clinically severe cases contributed to determine this uncertainty and represented an important limitation to a real estimate of influenza A H1N1 incidence in the pediatric population.

Information management was and remains a basic principle in the strategy to face a pandemic. During the last influenza epidemic information was ambiguous and not truthful. Messages conveyed by the mass media, however, emphasized virus pathogenicity and contributed to the adoption of important preventive measures such as handwashing both among the general population and healthcare workers.

## Abbreviations

P.E.R: Pediatric Emergency Room; I.L.I: Influenza Like Illness

## Competing interests

The authors declare that they have no competing interests.

## Authors' contributions

VT, VG and LN equally contributed to the study and to the draft of the paper. All authors read and approved the final manuscript.
